# Equivalent outcomes without increased morbidity in MPFL reconstruction and distalising tibial tubercle osteotomy versus isolated MPFL reconstruction

**DOI:** 10.1016/j.jor.2026.03.011

**Published:** 2026-03-08

**Authors:** Kimberley Kai Lun, Yoong Ping Lim, Jonathan Warnock, David A. Parker

**Affiliations:** a Sydney Orthopaedic Research Institute, St Leonards, NSW, Australia; b Sydney Local Health District, Camperdown, NSW, Australia; c The University of Sydney, Camperdown, NSW, Australia; d Musgrave Park Hospital, Belfast, United Kingdom; e Charles Darwin University, Brinkin, NT, Australia

**Keywords:** Patellar instability, Patellar dislocation, Medial patellofemoral ligament reconstruction, Tibial tubercle osteotomy

## Abstract

**Importance/aims:**

Isolated MPFL reconstruction (MPFLR) and, concomitant MPFLR with tibial tubercle osteotomy (MPFLR + TTO) are common operations for recurrent patellar instability. The addition of a TTO procedure has been associated with higher patient morbidity, slower recovery and a greater complication risk(Payne et al., 2015) .11 This study aimed to investigate the post-operative mid to long-term clinical outcomes of MPFLR compared with MPFLR and concomitant distalising TTO performed for patella alta.

**Methods:**

Patients from a specialist orthopaedic clinic who underwent an MPFLr ± TTO between 2006 and 2020 were contacted to report their clinical and functional outcomes after at least 12 months post-op. 70 patients consisting of 73 operated knees, responded. Caton-Deschamps index, sulcus angle, TT-TG and patellar tilt were assessed from pre-op MRIs by two independent reviewers. Statistical analysis was performed to compare post-operative outcomes and radiographic measurements between groups.

**Results:**

There was no difference in post-operative outcome measures, re-dislocation rate or rate of return-to-sport between cohorts. Both cohorts had a high preoperative incidence of patellar alta, trochlear dysplasia and patella tilt. The MPFLR + TTO cohort had a greater preoperative patellar height and greater degrees of patellar tilt than the MPFLR cohort.

**Conclusion:**

While an additional TTO is known to be associated with a higher post-operative morbidity risk, in the present study, both cohorts had equivalent post-operative outcomes without increased morbidity. Patients with anatomical variants such as greater patellar height may benefit from an additional TTO in the mid-to long-term.

**Level of evidence:**

Level III

**What are the new findings:**

• There was no difference in post-operative outcome measures, re-dislocation rate or return-to-sport between patients who underwent an isolated MPFLR versus MPFLR + TTO.
• Patients with higher degrees of patellar alta may benefit from an additional distalising TTO, resulting in comparable post-operative outcomes without increased morbidity in the mid to long-term.

## 1 Introduction

Recurrent patellar instability is a common, disabling condition that can necessitate surgical intervention. Medial patellofemoral ligament reconstruction (MPFLR) is a commonly used surgical procedure for patellofemoral instability.^1^ Further combining an MPFLR with an additional tibial tubercle osteotomy (TTO) can also be appropriate for selected patients^1^, ^2^, ^3^. While successful clinical outcomes for combined MPFLR and TTO procedures are well established in the literature 4, 5, 6, the addition of a TTO procedure is associated with higher patient morbidity, slower recovery and a greater risk of complications.^7^

Ongoing debate exists regarding the indications for an additional TTO in recurrent patellar instability. The addition of a TTO has been conventionally recommended for patients with specific osseous anatomical variants. Franciozi, Ambra, Albertoni, Debieux, de Mello Granata Jr, Kubota, Carneiro, Abdalla, Luzo and Cohen^3^ and Kim, Sim, Yang, Kim, Wang and Seon^8^ both found patients with an increased tibial tubercle to trochlear groove distance had better functional and clinical outcomes when undergoing combined MPFLR and TTO procedures, compared to an MPFLR alone. Allen, Krych, Johnson, Mohan, Stuart and Dahm^9^ reported low rates of recurrent instability in patients with trochlear dysplasia who underwent an additional TTO procedure. Conversely, several studies have reported good outcomes in patients undergoing isolated MPFLR, irrespective of bony pathologies such as increased tibial tubercle to trochlear groove distance, patellar alta and trochlea dysplasia.^10^^,^^11^

This paper aimed to investigate the post-operative mid to long-term clinical outcomes of MPFLR compared with MPFLR and concomitant distalising TTO. We also aimed to compare pre-operative radiological measurements between MPFLR patients with and without concomitant TTO (MPFLR + TTO), to determine radiographic features correlated with improved post-operative outcomes. We hypothesised that clinical outcomes between MPFLR and MPFLR + TTO patients would be equivalent in the mid to long term.

## 2 Methods

### 2.1 Patient selection

This was a retrospective cohort study of patients who underwent MPFL reconstruction with or without a tibial tubercle osteotomy (TTO) at a private orthopaedic clinic between 2006 and 2020. Patients were included if they had a history of at least one documented patellar dislocation, and subsequently underwent patellar stabilisation surgery on the same leg. Patellar stabilisation surgery was either an MPFLR, or an MPFLR + TTO. The addition of a distalising TTO was indicated in patients with patella alta. All patients were assessed by a senior orthopaedic surgeon to determine their operative plan. All TTO procedures involved distalisation of the tibial tubercle.

Patients were excluded if had undergone previous surgery on the affected knee or underwent a procedure that was not an MPFLR or MPFLR + TTO. Patients were also excluded if they were skeletally immature at the time of operation, had congenital disease contributing to their presentation or were less than 12 months post-operation at the time of data collection.

### 2.2 Surgical Technique

#### 2.2.1 MPFL reconstruction

A midline longitudinal incision was made along the medial border of the tibial tubercle. Dissection was performed to expose the pes anserine tendons and a hamstring tendon graft was stripped from the muscular tendinous junction. A whipstitch was tied at either end and stored in the wound.

Two short incisions were made over the medial patella and medial femoral condyle to identify the attachment site for the MPFL reconstruction. K-wires were placed at the proposed sites and checked with fluoroscopy.

Two corkscrew anchors were used to secure the hamstring tendon onto the proximal one third of the patella. The graft was then passed between the retinaculum and synovium to the posterior wound. A guidewire was then passed from the previously located position out through the anterolateral thigh and overdrilled until the lateral cortex, and then drilled through the lateral cortex. The two tendons were then delivered into the tunnel.

After the completion of a TTO, if it was to be performed, the MPFL graft was fixed on the femoral side. The patella was held central with the knee at 30° flexion. A screw was placed posteriorly in the tunnel. The knee was moved through a full range of motion and any lateral excursion of the patellar was assessed.

#### 2.2.2 Tibial tubercle osteotomy

An anteromedial longitudinal incision was made along the length of the tibial tubercle. Dissection exposed the tibial tubercle. A transverse, slightly bevelled cut was made distally and a distal portion was removed to allow distalisation of the tubercle. The osteotomy was fixed to the bony bed using two fully threaded bicortical countersunk screws. Position was then checked with fluoroscopy and fixation was checked by ranging the knee from full extension to 130 degrees of flexion.

### 2.3 Rehabilitation protocol

Patients who underwent an isolated MPFLR were placed in an extension brace and allowed to weight bear immediately post-operatively. Patients then progressed through gradual advancement of knee flexion: from 0 to 90° at 1 week post-op, then 0-115° at 2 weeks post-op, and then 0-120° at 3-6 weeks post-op.

Patients who underwent an additional TTO had a 6-8 week non-weight bearing period in extension bracing. After 6-8 weeks, weight bearing was gradually introduced alongside range of motion and strengthening exercises.

### 2.4 Data collection

Eligible patients were contacted via text and phone call to complete Kujala and Norwich Patellar Instability questionnaires and report any ongoing discomfort. Patients were also asked to report their return to sport, and any further surgeries or non-traumatic patellar dislocations since their operation.

**Fig. 1:**
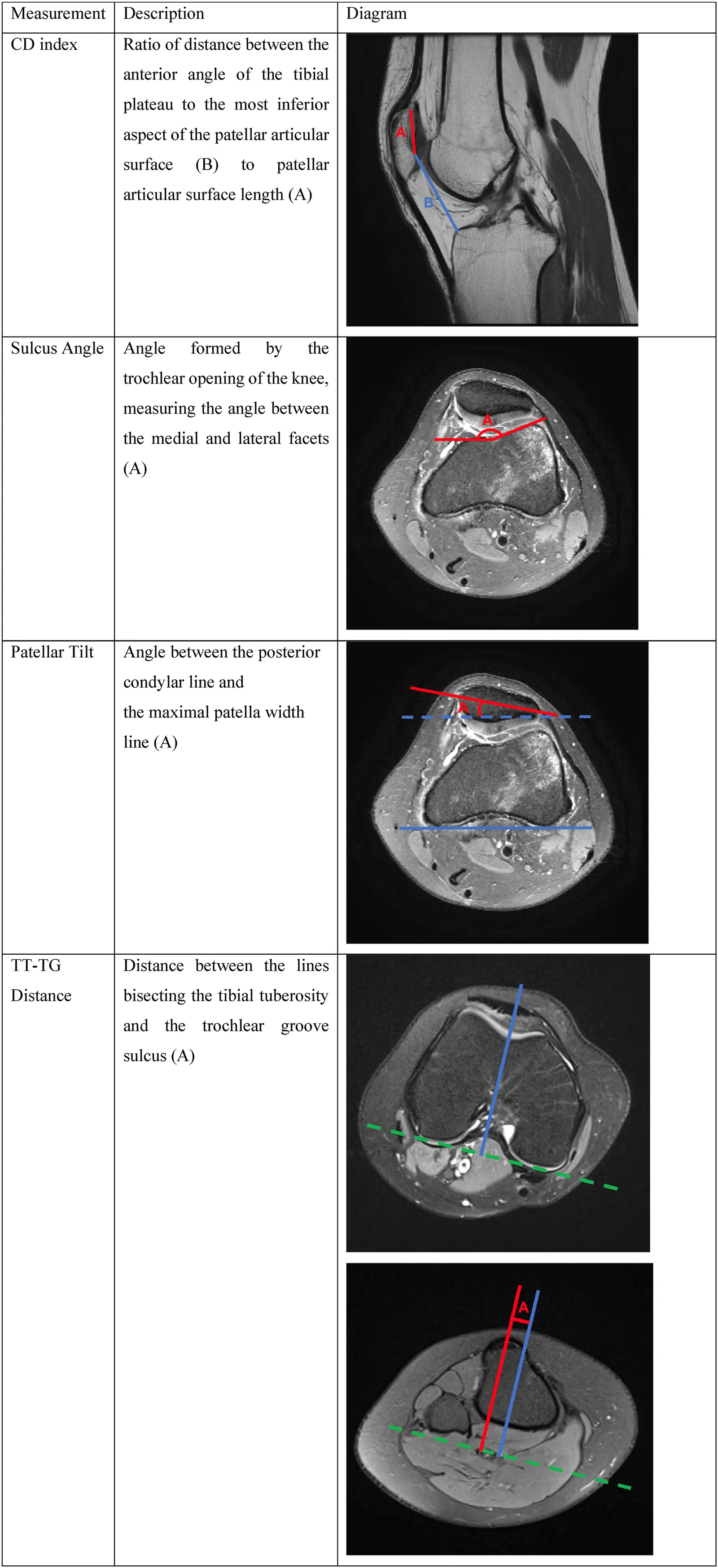
Radiographic measurements.

All patients underwent preoperative magnetic resonance imaging (MRI) of the affected knee. Radiographic measurements of Insall-Salvati ratio,^12^ Caton-Deschamps index,^13^ Blackburne-Peele ratio,^14^ sulcus angle, tibial tubercle – trochlear groove (TT-TG) distance^15^ and patellar tilt were assessed from pre-operative MRIs by two independent doctors. The radiographic measurements are outlined in Fig. 1.

### 2.5 Statistical analysis

The statistical language SPSS was used for analysis. All continuous variables were compared using independent t-tests, and all categorical variables were compared between groups using Chi-square tests. A significance level of 5% was used throughout.

## 3 Results

**Fig. 2:**
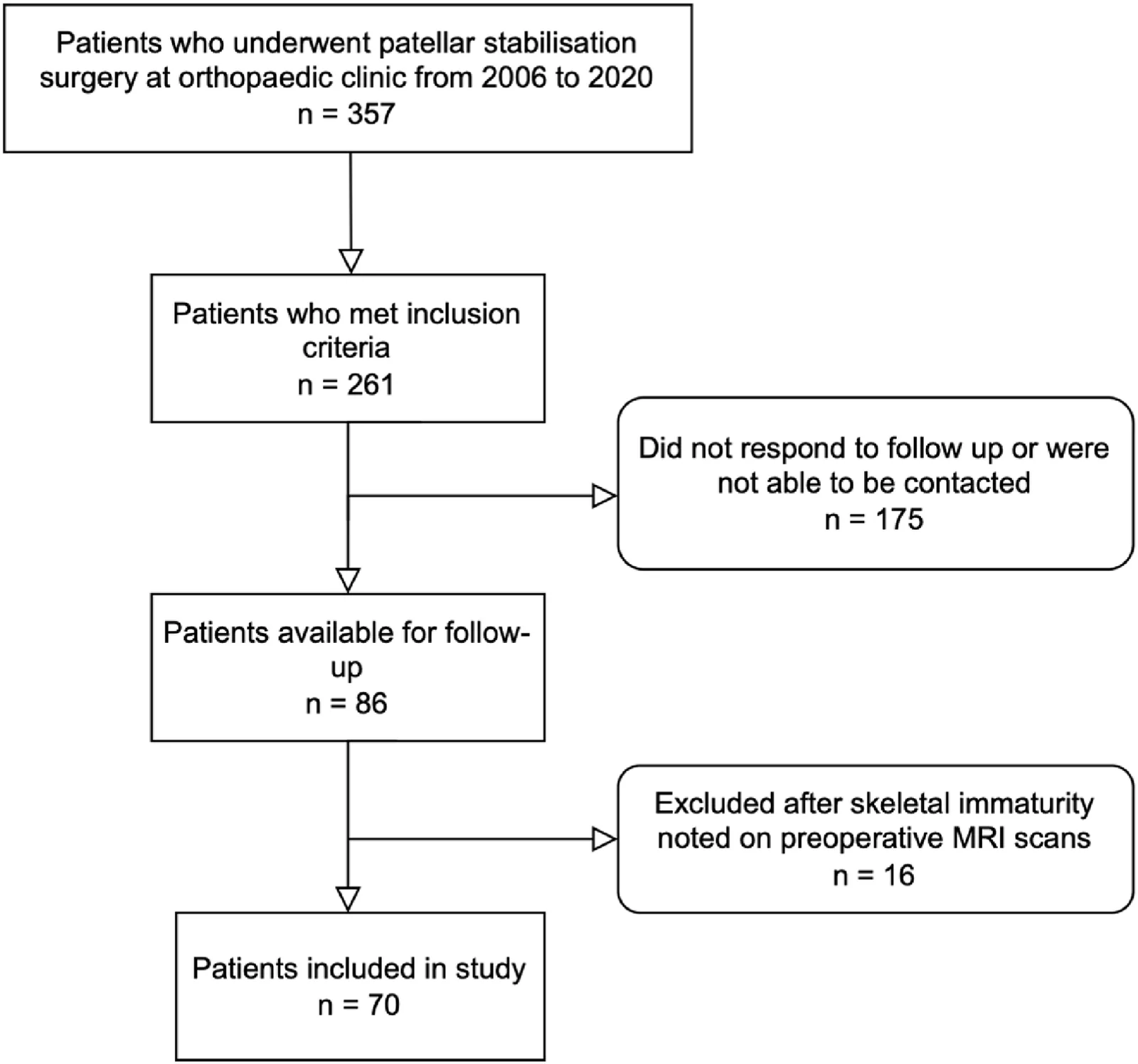
Patient inclusion.

Between 2006 and 2020, 357 patients (378 knees) underwent patellar stabilisation surgery for recurrent patellar instability. After the inclusion criteria were applied and patients contacted, 70 patients consisting of 73 operated knees were included in this study (Fig. 2). Forty-four patients, consisting of 45 operated knees, underwent MPFLR, and 26 patients consisting of 28 operated knees, underwent MPFLR + TTO.

Pre-operative MRIs were available for 21 knees that underwent an isolated MPLFR and 16 knees that underwent a MPFLR + TTO.

### 3.1 Demographics

There was no significant difference between groups in demographic factors. Both cohorts were predominantly female with an average age of 25-30 years (Table 1). Patients were contacted between 1 and 15 years post procedure with an average follow up time of 5 years.

**Table 1:** Cohort demographics.

Characteristic	Mean ± SD (Range); n (%)		p-value
	MPFLR N = 45	MPFLR + TTO N = 28	
Age	25.8 ± 8.5 (13 – 44)	29.9 ± 9.6 (15 – 52)	0.059
Gender (Female)	31 (68.9%)	20 (71.4%)	0.818
Years post-surgery	5.0 ± 3.2 (1.3 – 15)	5.2 ± 2.6 (1.0 – 11.8)	0.862
Leg (Right)	18 (40.0%)	10 (35.7%)	0.446
Mechanism of injury^a^			0.499
Not noted	5 (11.1%)	4 (14.3%)	
Non contact	38 (84.4%)	24 (85.7%)	
Contact	2 (4.4%)	0 (0.0%)	
Contralateral patellar dislocations (Yes)	8 (17.8%)	6 (21.4%)	0.700

a Contact or non-contact mechanism of injury of index patellar dislocation.

### 3.2 Patient reported outcomes

There was no difference in pain, instability, return to sport, re-dislocation rate or reported discomfort between MPFLr versus MPFLR + TTO cohorts (Table 2, Table 3).

**Table 2:** Patient reported outcome measurements between groups – pain and instability.

Patient Reported Outcomes	Mean ± SD		P-value
	MPFLr N = 45	MPFLr + TTO N = 28	
Kujala Score^a^	85.88 ± 13.10	83.44 ± 14.98	0.485
Norwich Patellar Instability Score^b^	38.38	32.11	0.608

a Kujala score is a 13-item tool for patellofemoral pain graded from 0 to 100 with 100 indicating no pain and 0 indicating the maximum amount of pain.

b Norwich Patellar Instability score is a 19-item tool evaluating the impact of patellofemoral instability on joint function with 0 indicating no instability, and a maximum score of 225 indicating maximal patellofemoral instability.

**Table 3:** Patient reported outcome measurements between groups.

Outcome	MPFLr N = 45	MPFLr + TTO N = 28		p-value
Re-dislocation rate	3 (6.7%)	1 (3.6%)		0.572
Revision surgery rate	2 (4.4%)	0 (0%)		N/A
Return to sport rate^a^	70.5%	71.4%		0.678
Reported discomfort (Yes)	29 (64.4%)		19 (67.9%)	0.765

a Successful return to sport defined as return to previous activity levels.

Three patients in the MPFLR cohort and three patients in the MPFLR + TTO cohort had since had a patella re-dislocation on the operated leg. Two patients in the MPFLR cohort had a further procedure – one patient underwent a revision MPFL reconstruction and one patient declined to report the type of procedure they underwent. One patient in the MPFLR + TTO cohort had a further knee arthroscopy.

In the MPFLR cohort, 29 (64%) patients reported ongoing discomfort with 20 of these patients reporting anterior knee pain as the source of discomfort. In the MPFLR + TTO cohort, 19 (68%) patients reported ongoing discomfort with 12 of these patients reporting anterior knee pain as the source of discomfort.

### 3.3 Preoperative radiographic measurements

Preoperatively, both cohorts had a high average patellar height, and greater degrees of trochlear dysplasia and patella tilt. Between groups, patients in the MPFLR + TTO cohort had greater patellar height as measured by the Caton-Deschamps index (p < 0.05) and increased patellar tilt (p < 0.05) compared with the MPFLR cohort (Table 4).

**Table 4:** Radiographic measurements between groups.

Measurement	Mean ± SD		P-value
	MPFLr N = 21	MPFLr + TTO N = 16	
Caton-Deschamps index (Patella alta >1.3)	1.20 ± 0.13	1.27 ± 0.17	**0.003**
Sulcus Angle (Trochlear dysplasia >145-150)	147.84 ± 8.43	149.48 ± 7.74	0.533
Patellar Tilt^a^ (Patellar tilt >11)	17.95 ± 8.67	21.16 ± 9.35	**0.025**
Tibial Tubercle-Trochlear Groove Distance^b^ (Patellar translation >20)	13.45 ± 5.23	14.34 ± 4.47	0.773

a N = 28 in MPFLr cohort, and N = 24 in MPFLr + TTO cohort.

b N = 29 in MPFLr cohort, and N = 27 in MPFLr + TTO cohort.

## 4 Discussion

In this study, there were no differences between MPFLR and MPFLR + TTO cohorts in post-operative pain, instability, re-dislocation rate, revision surgery or return-to-sport at an average of 5 years of follow-up. The low rates of re-dislocation or revision surgery are consistent with the literature.^16^

While the increased morbidity associated with bony realignment procedures has deterred some surgeons from the addition of a TTO,^17^^,^^18^ our study found comparable post-operative outcomes between the two cohorts. In our clinical practice, a TTO is indicated for the tibial tubercle distalisation in patients with patella alta, as reflected by the greater preoperative patellar height in the MPFLR + TTO cohort. Patients in the MPFLR + TTO cohort also had greater average sulcus angles, patellar tilts and TT-TG distances than those in the MPFLR group. These observations support osseous patellar malalignment and indicate a concomitant TTO. Patellar alta is a well-established risk factor for recurrent patellar instability, providing a rationale for tibial tubercle distalisation to improve osseous restraint with earlier patellar-trochlea engagement during knee flexion.^5^^,^^19^ Our clinical practice is consistent with the results reported by Sappey-Marinier, Sonnery-Cottet, O'Loughlin, Ouanezar, Reina Fernandes, Kouevidjin and Thaunat^20^ who noted that a CD index greater than 1.3 was a predictive factor for isolated MPFLR failure.

The findings of this study agree with recent literature. Markus et al.^21^ found comparable functional outcomes, pain scores and patient satisfaction in patients who underwent MPFLR, and those who underwent MPFLR and anteromedialising TTO in a matched population of 118 patients 4 years post-operatively. Similarly, in a prospective study of 126 patients at an average of 5 years follow-up, Neri et al.^22^ found that MPFLR, whether isolated or combined with a distalising TTO, improves long-term clinical results.

Some studies have described satisfactory outcomes of isolated MPFLR irrespective of increased TT-TG distance, with relatively short follow-up post-operatively (i.e. less than 2 years).^10^^,^^11^ In a case series of 90 patients, Eriksson et al.^11^ found isolated MPFLR produced significant improvement in patient-reported outcome scores between 1 and 2 years post-operatively, irrespective of bony pathologies such as increased TT-TG distance, CD index or trochlea dysplasia. Similarly, Matsushita et al.^10^ found equivalent patient-reported outcomes in patients with a TT-TG distance greater than 20 and those less than 20 who underwent an isolated MPFLR at one year post-operative. Contrary to these results, increased TT-TG distance was not an indication for an additional TTO in our cohort, and these equivalent outcomes between MPFLR and MPFLR + TTO cohorts, irrespective of TT-TG distance, persist in the mid to long term.

While self-reported pain and instability were low in both cohorts, discomfort was reported by more than 60% of both MPLFR and MPLR + TTO patients. Anterior knee pain was the leading reported cause of discomfort. This suggests a degree of ongoing pain endured by both cohorts post-operatively that is not detected on conventional patient-reported outcome measures. Further investigation into the sensitivity of existing patient-reported outcome measures is required to further quantify this finding.

## 5 Limitations

This study was limited by poor follow-up rates. Only 27% of eligible patients responded to follow-up, and among those who did, only 51% of included patients had a pre-operative MRI available for review, introducing selection bias. Secondly, no strict threshold for patella height was used to indicate a TTO, as recruitment was from multiple surgeons over a long retrospective period. However, there were significant differences in patella height between cohorts, suggesting patella alta as a factor in guiding surgical candidacy. Thirdly, while return to sport rates were equivalent between groups, the timing of return to sport was not reported. Based on each surgeon's post-operative protocol, the time to return-to-sport may vary. Finally, as a retrospective study, there were no baseline pre-operative patient-reported outcomes to compare to postoperative outcomes.

## 6 Conclusion

While both isolated MPFLR and MPFLR + TTO have demonstrated excellent patient outcomes, contention still exists over the optimal surgical selection criteria. In this study, the subset of patients undergoing an additional TTO demonstrated comparable outcomes without additional morbidity, to patients undergoing a less invasive isolated MPFLR. Patella alta and patellar tilt were more common in the cohort undergoing TTO, and the equivalent outcomes in these patients suggested these factors were appropriate indications for TTO and may serve as valid indications for TTO in this population. Further study into the impact and interaction of radiographic and demographic factors in post-operative outcomes, will help define more precise thresholds for this additional procedure.

## Consent to publish

Participant provided written consent for publication of images.

## Informed consent

Informed consent was obtained from all individual participants included in the study. If the participant was not of legal age to provide independent consent, both parental consent and child assent were obtained prior to data collection.

## Ethical approval

The study design was approved by the Human Research Ethics Committee of Northern Sydney Local Health District (NSLHD reference: RESP/17/110).

## Authors’ contributions

Conceptualization: Kimberley Kai Lun, Yoong Ping Lim, Jonathan Warnock,

Project administration and Supervision: David A. Parker.

Investigation, Formal analysis and Validation: Kimberley Kai Lun, Yoong Ping Lim, Jonathan Warnock.

Writing original draft: Kimberley Kai Lun.

Writing-review and editing: Kimberley Kai Lun, Yoong Ping Lim, Jonathan Warnock, David A. Parker.

## Funding

This study received no funding.

## Declaration of competing interest

Kimberley Kai Lun – no conflict of interest.

Yoong Ping Lim – associate editor (J. Orthopaedic Research Surgery and Minerva Orthopaedic).

Jonathan Warnock – no conflict of interest.

David A. Parker- Paid consultant and Speaker (Smith and Nephew), Journal editorial board (JISAKOS, AJSM, OJSM and AP-smart) and shareholder (Ganymed Robotics and Personalized Surgery).
